# Complete chloroplast genome characterization and phylogenetic analysis of *Clematis mandshurica* (Ranunculaceae)

**DOI:** 10.1080/23802359.2022.2073839

**Published:** 2022-05-10

**Authors:** Yi Cui, Lihua Yang, Yanzhe Ding, Yingxin Sun, Jiao Wang, Yunfei Xi, Mei Han, Limin Yang, Zhongming Han, Yunhe Wang

**Affiliations:** a College of Chinese Medicinal Materials, Jilin Agricultural University, Changchun, China; b State Key Laboratory of JLP-MOST for Ecological Restoration and Ecosystem Management, Changchun, China; c College of Life Science, Changchun Sci-Tech University, Changchun, China

**Keywords:** *Clematis mandshurica*, chloroplast genome, phylogenetic analysis

## Abstract

The complete chloroplast genome sequence of *Clematis mandshurica* Ruprecht (1867), a specie of the Ranunculaceae family, and its phylogenetic relationships with other species have been reported in this study. The complete chloroplast genome of *C. mandshurica* is 159,563 bp in length, including a large single-copy (LSC) region of 79,360 bp, a small single-copy (SSC) region of 18,121 bp, and a pair of identical inverted repeat regions (IRs) of 31,041 bp. The genome encodes a total of 132 genes, including 90 protein-coding genes, 34 transfer RNA (tRNA) genes, and eight ribosomal RNA (rRNA) genes. The phylogenetic analysis reveals that *C. mandshurica* was found to be closest to *Clematis taeguensis*. The complete chloroplast genome of *C. mandshurica* contributes to a better understanding of phylogenetic relationships among *Clematis* species.

*Clematis* is a cosmopolitan genus of about 355 species in the Ranunculaceae (Wang and Li 2005; Yang et al. 2019; Chen et al. 2021), which is widely distributed throughout the world. *Clematis mandshurica* root is the most famous traditional Chinese herbal medicines obtained from *Clematis* species (Hao et al. 2013), widely used as Wei Ling Xian in traditional medicine (Lee et al. 2014) and is native to China, Korea, Mongolia, and the Russian Far East (Dong et al. 2016). The dried roots and rhizomes of *C. mandshurica* have been used as an analgesic, anti-inflammatory, anti-tumor, and dispel wind agent (Huang 1999; He et al. 2011; Ling et al. 2013; National Pharmacopoeia Committee 2020). At present, the research on *C. mandshurica* mainly focused on cultivation techniques (Han et al. 2011, 2021), chemical constituents and pharmacological activities (Lee et al. 2020; Lin et al. 2021). However, the genetics and molecular biology of *C. mandshurica* are poorly understood. Therefore, we here report the first complete chloroplast genome of *C. mandshurica* by high throughput sequencing technology, which will provide valuable bioinformatic data for understanding the systematics of *C. mandshurica* and genetic research.

Fresh leaves of *C. mandshurica* were provided from Medicinal Herb Garden, Jilin Agricultural University, Changchun, China (43°48′23″N, 125°24′57″E). The voucher specimen was deposited in the Herbarium of College of Chinese Medicinal Materials, Jilin Agricultural University (https://zhongyao.jlau.edu.cn; Zeliang Lü, lvzeliang@foxmail.com) under the voucher number Y. Cui 2021009. Genomic DNA was extracted by a QIAquick Gel Extraction kit (Qiagen, Hilden, Germany). Pair-end raw reads were obtained by PE 150 library and the Illumina Hiseq 2500 platform. Finally, the raw data (1.1 Gb) were obtained. Genome assembly and annotation were conducted using metaSPAdes9 (Nurk et al. 2017) and CPGAVAS2 (Shi et al. 2019), respectively. The annotated cp genome sequence was submitted to GenBank under the accession number OK375873.

The length of complete chloroplast genome of *C. mandshurica* was 159,563 bp, displaying a large single-copy (LSC), a small single-copy (SSC), and a pair of inverted repeat (IR) regions of 79,360 bp, 18,121 bp, and 31,041 bp, respectively. A total of 132 genes were annotated, including 90 protein-coding genes, 34 transfer RNA (tRNA) genes, and eight ribosomal RNA (rRNA) genes. The GC content of the cp genome was 37.98%, and the GC content of the LSC, SSC, and IR regions were 36.3%, 31.3%, and 42.1%, respectively.

The maximum-likelihood (ML) phylogenetic tree was generated based on the complete genome of *C. mandshurica* and other species of the Ranunculaceae (Figure 1). The 20 complete chloroplast sequences were aligned using MAFFT software (Katoh and Standley 2013), and a phylogenetic tree was constructed using MEGAX (Kumar et al. 2018) with a generalized time-reversible (GTR) sequence evolution model and an ML for tree improvement. *Clematis mandshurica* was found to be closest to *Clematis taeguensis* in tribe Anemoneae, subfamily Ranunculoideae. The phylogenetic analysis resolved great chloroplast divergence within the genus *Clematis* (Kyun et al. 2021). In conclusion, the complete chloroplast genome of *C. mandshurica* contributes to a better understanding of the phylogenetic relationships among *Clematis* species, which can contribute important information for understanding phylogenetic and evolutionary studies of Ranunculaceae.

**Figure 1.**
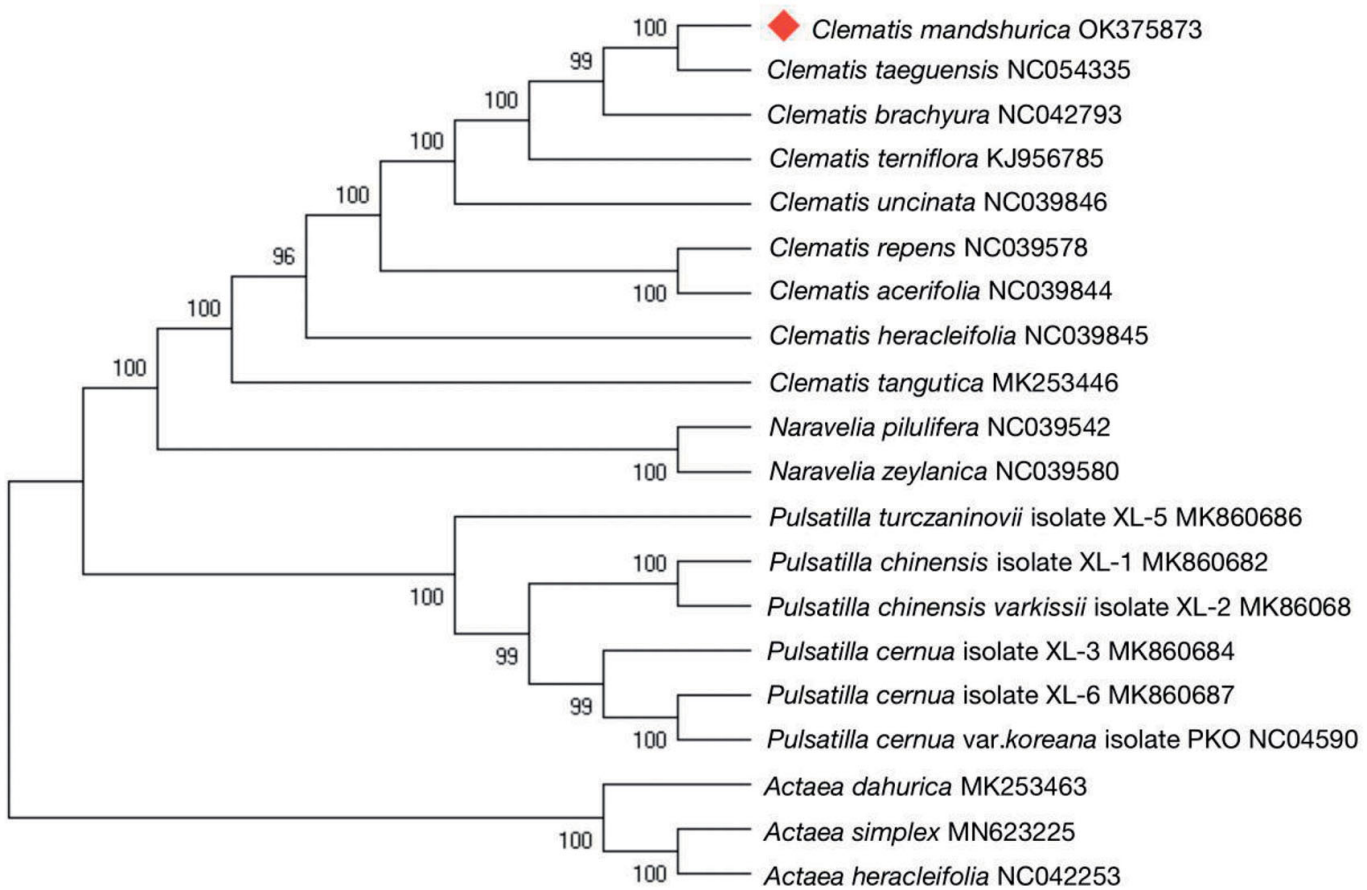
The ML tree based on the cp genome of *Clematis mandshurica* and other 19 species that were downloaded from GenBank and Anemone as the outgroup. The numbers on the branches are bootstrap values.

## Ethical approval

The materials used in this study are not included in IUCN red list, the collection area is not a protected area. Research and collection of plant material was conducted according to the guidelines provided by JLAU (Jilin Agricultural University). Permission was granted by the China Biotechnology Center and Science and Technology Department of Jilin Province.

## Author contributions

Zhongming Han, Limin Yang, and Yunhe Wang: conceptualized and designed research; Yi Cui, Lihua Yang, and Mei Han: analyzed data and wrote original draft of the manuscript; Yanzhe Ding, Yingxin Sun, Jiao Wang, and Yunfei Xi: contributed to research materials and to the draft manuscript. All authors read and approved the final manuscript.

## Data Availability

The genome sequence data that support the findings of this study are openly available in GenBank of NCBI (https://www.ncbi.nlm.nih.gov/) under the accession number OK375873. The associated BioProject, SRA, and Bio-Sample numbers are PRJNA764578, SRR16071938, and SAMN21841556, respectively.
